# I Meant to Do That: Determining the Intentions of Action in the Face of Disturbances

**DOI:** 10.1371/journal.pone.0137289

**Published:** 2015-09-01

**Authors:** Justin Horowitz, James Patton

**Affiliations:** 1 Bioengineering, University of Illinois at Chicago, Chicago, Illinois, United States of America; 2 Sensory Motor Performance Program, Rehabilitation Institute of Chicago, Chicago, Illinois, United States of America; University Medical Center Goettingen, GERMANY

## Abstract

Our actions often do not match our intentions when there are external disturbances such as turbulence. We derived a novel modeling approach for determining this motor intent from targeted reaching motions that are disturbed by an unexpected force. First, we demonstrated how to mathematically invert both feedforward (predictive) and feedback controls to obtain an intended trajectory. We next examined the model’s sensitivity to a realistic range of parameter uncertainties, and found that the expected inaccuracy due to all possible parameter mis-estimations was less than typical movement-to-movement variations seen when humans reach to similar targets. The largest sensitivity arose mainly from uncertainty in joint stiffnesses. Humans cannot change their intent until they acquire sensory feedback, therefore we tested the hypothesis that a straight-line intent should be evident for at least the first 120 milliseconds following the onset of a disturbance. As expected, the intended trajectory showed no change from undisturbed reaching for more than 150 milliseconds after the disturbance onset. Beyond this point, however, we detected a change in intent in five out of eight subjects, surprisingly even when the hand is already near the target. Knowing such an intent signal is broadly applicable: enhanced human-machine interaction, the study of impaired intent in neural disorders, the real-time determination (and manipulation) of error in training, and complex systems that embody planning such as brain machine interfaces, team sports, crowds, or swarms. In addition, observing intent as it changes might act as a window into the mechanisms of planning, correction, and learning.

## Introduction

Disturbances, distractions, and pathologies can interfere in many situations and prevent our actions from matching our intent. A pilot, for example, may fail to complete a maneuver because of turbulence. Such challenges are broadly present in many human-machine interactions. One may speculate on how we might use these very same machines to elucidate the underlying intention. Such a possibility would be broadly useful in any area where intended actions might be thwarted by disturbances.

It is not necessary for the brain to represent intent explicitly to generate action. Instead the nervous system might simply learn the relationship between muscle activations and the accomplishment of goals. For example, one might simply evaluate a motion outcome and adjust descending signals to the muscles. At the same time, one may not deny the fact that there are tasks that require an explicit representation of a trajectory, such as performing a dance, drawing a picture, or conducting an orchestra. For these tasks, intent is likely represented at some level in the nervous system. Nevertheless, the mechanics of the body dictate the existence of an equilibrium whether intent is explicitly represented or not. Here our goal was to answer a modest question, “*when* movement intent is a changing equilibrium, can we recover it despite external disturbances?”

The words *intent* and *equilibrium* are contentious because of the various definitions that exist in the literature (S1 Text). Our simple definition here is that the intent is the path that would have been taken had there been no external disturbances. In other words, action will match intent in the absence of any unexpected disturbances. We take intent to be a dynamic *equilibrium* when action approaches intent over time following any sufficiently small disturbance.

Dynamic models that strive to understand intent are wide ranging, and can include mathematical models that range in application from swarm prediction to athletic performance. The plant (such as the actions of a crowd of people or the motions of an opposing team) may be too complex to model with simple linear transformations as we present above. However, by substituting any approximation or lookup table of input-outcome tendencies, it is possible in a variety of applications to obtain the intent, even when disturbed. In other words, it may be possible to intervene before a car crashes or an opposing team scores by knowing the intent behind their action.

Here we propose a general method, *intent extraction*, which calculates the intended trajectory even in the face of disturbances by relating environmental interaction and state to intent. The method is a class of filters that can infer the intended trajectory from the disturbance (turbulence), process (dynamic equations), actuator (muscles), and motion. The method affords new ways to study models of motor control, understand the timing and composition of intent, and how it might be altered by disturbances and injuries. Below we first present the mathematics underlying intent extraction. Then, for simplicity in this initial study, we create synthetic reaching data to examine the quality and uncertainty of this method. Finally, we demonstrate its effectiveness extracting intended action from real human reaches that have been disrupted by unpredictable disturbances. We demonstrate this effectiveness by testing the hypothesis that disturbance does not change intended action for at least 120 milliseconds. This early experiment also provides some new insights on how intended trajectories can change in reaction to disturbances.

## Methods

The sections below describe the theoretical method used and the means by which we evaluated it. First, we present an idealized system using synthetic data that demonstrated the concept and provided an understanding of the computational process. Next, we present an experimental study on humans that evaluated success in a simple test of the approach’s ability to extract straight intentions even when disturbed.

### Intent Extraction: Deducing the Desired Trajectory

We first demonstrate the *intent extraction* approach in human motor control, but we later show that the process is applicable to any controlled process with an invertible highest order plant term. The process begins by presuming a model of the controller. Here, we choose the well-known motion control structure of Shadmehr and Mussa-Ivaldi [1] where the feedforward aspect of the controller perfectly predicts the plant and linearizes the system through cancellation. Additionally, linear feedback rejects position and velocity error. This model was chosen to help illuminate the approach, but later we present how to generalize this approach to more complex models without loss of generality. The equation governing the passive planar dynamics (plant) of musculoskeletal structure is of the form,

$$\underset{\text{Plant}}{\underset{︸}{\overset{\text{Inertia}}{\overset{︷}{M\left(q\right)\ddot{q}}}+\overset{\text{Coriolis,}\;\text{Centripetal}}{\overset{︷}{G(q,\dot{q})}}}}+E=0$$ (1)

where *M* is the mass matrix, *q* is the joint angles, $\dot{q}$ is joint angular velocity, $\ddot{q}$ is joint angular acceleration, *G* contains both Coriolis and centripetal effects, and *E* is any externally-applied torque. The motion behavior changes with the addition of feedforward and/or feedback controllers,

$$\underset{\text{Plant}}{\underset{︸}{\overset{\text{Inertia}}{\overset{︷}{M\left(q\right)\ddot{q}}}+\overset{\text{Coriolis,}\;\text{Centripetal}}{\overset{︷}{G(q,\dot{q})}}}}+E=\underset{\text{Feedforward}\;\text{Controller}}{\underset{︸}{\overset{\text{Inertia}}{\overset{︷}{\hat{M}\left(q_{d}\right){\ddot{q}}_{d}}}+\overset{\text{Coriolis,}\;\text{Centripetal}}{\overset{︷}{\hat{G}\left(q_{d},{\dot{q}}_{d}\right)}}+\hat{E}}}+\underset{\text{Impedance,}\;\text{Feedback}\;\text{Controller}}{\underset{︸}{K_{p}\left(q_{d}-q\right)+K_{d}\left({\dot{q}}_{d}-\dot{q}\right)}}$$ (2)

where terms with hats over them ($\hat{M}$, $\hat{G}$, or $\hat{E}$) indicate that they represent the nervous system’s best estimate of the forces and dynamics it will encounter, which is also known as an internal model [1]. This portion of the system serves as an inverse-dynamics feedforward controller that cancels out the dynamics of the arm in the torque balance. If the nervous system has sufficient experience and is expecting *E*, it is included as part of the internal model, $\hat{E}$; otherwise $\hat{E}$ is set to zero. *K*_*p*_ and *K*_*d*_ are the lumped impedance and feedback terms that employ a moving state equilibrium to accomplish the desired trajectory, *q*_*d*_. This *q*_*d*_ has a dual meaning in that it signifies both the unknown desired trajectory that we seek to discover and also the moving equilibrium trajectory of the arm.

For typical dynamic simulations, in order to determine the trajectory of the system (i.e., the forward dynamics problem), this second-order differential equation is solved by numerical integration to determine the solution to the initial value problem in time. This entails algebraic manipulation to solve for $\ddot{q}$, followed by integration to determine the state trajectory. Intent determination takes the novel approach of instead solving for ${\ddot{q}}_{d}$,

$${\ddot{q}}_{d}=\hat{M}{\left(q_{d}\right)}^{-1}\{M\left(q\right)\ddot{q}+G\left(q,\dot{q}\right)+E-\left[\hat{G}\left(q_{d},{\dot{q}}_{d}\right)+\hat{E}+K_{p}\left(q_{d}-q\right)+K_{d}\left({\dot{q}}_{d}-\dot{q}\right)\right]\}$$ (3)

such that ${\ddot{q}}_{d}$ can then be integrated numerically using a differential equation solver to determine the intended state trajectory *q*_*d*_(*t*). This relies on many assumptions: The model of plant and controller must be accurate and precise. The initial conditions must be available and accurate. The mass matrix estimate, $\hat{M}$, must be invertible. Externally-applied force must be precisely and accurately measured. If all of these conditions are met, then the system yields an accurate estimate of the intent.

To explore the conditions under which this procedure for recovering the intent trajectory can be generalized, we take a series of steps. First, we distinguish between joint space and generalized space by introducing a generalized state variable, *x*. Second, we separate the plant (arm) into its process (dynamics), *P*, and actuator (muscles), *A*, components. Third, we model these components in a very general sense as operations,

$$\underset{\text{Plant}}{\underset{︸}{\overset{\text{Process}}{\overset{︷}{\sum_{n=1}^{N}P_{n}x^{\left(n\right)}}}+\overset{\text{Actuator}}{\overset{︷}{\sum_{m=1}^{M}A_{m}{\left(x-x_{e}\right)}^{\left(m\right)}}}}}+E=0$$ (4)

where *x*_*e*_ is the equilibrium trajectory of the actuator. Fourth, we describe a control law for the actuator using the same expansions,

$$\overset{\text{Internal Model}}{\overset{︷}{\sum_{n=1}^{N}{\hat{P}}_{n}x_{d}^{\left(n\right)}+\hat{E}}}+\overset{\text{Actuator}}{\overset{︷}{\sum_{m=1}^{M}A_{m}{\left(x_{d}-x_{e}\right)}^{\left(m\right)}}}=0$$ (5)

where an internal model, $\hat{P}$ and $\hat{E}$, predicts system dynamics and external disturbances in order to determine the actuator equilibrium *x*_*e*_ such that *x* will track a desired path *x*_*d*_. The feedforward component must use the actuator and hence it shares the actuator’s equilibrium with the plant. This allows us to finally relate the physical system and its control law by solving for *x*_*e*_ and substituting into Eq (4),

$$\overset{\text{Process}}{\overset{︷}{\sum_{n=1}^{N}P_{n}x^{\left(n\right)}}}+E=\overset{\text{Internal Model}}{\overset{︷}{\sum_{n=1}^{N}{\hat{P}}_{n}x_{d}^{\left(n\right)}+\hat{E}}}+\overset{\text{Feedback (Actuator)}}{\overset{︷}{\sum_{m=1}^{M}A_{m}{\left(x_{d}-x\right)}^{\left(m\right)}}}$$ (6)

wherein *x*_*e*_ vanishes recovering our familiar model. While a proper choice of *x*_*e*_ is perhaps essential for control, our approach does not require a model relating this equilibrium and intended trajectory. Note also that the actuator’s equilibrium *x*_*e*_ is not the process’s equilibrium unless all derivatives of *x*_*d*_ are zero.

The fact that the actuator’s equilibrium *x*_*e*_ is not the process’s equilibrium is important as the actuator state (λ) has been claimed to be the intended movement [2] (S1 Text). This upholds Gomi and Kawato’s finding that the arm muscles’ equilibrium does not represent reaching intent [3]. If the highest order coefficient of the dynamic model of the process ${\hat{P}}_{N}$ can be inverted, the impedances can be modeled, and the system is stable (e.g., the stiffness and damping are positive), it is possible to solve for $x_{d}^{\left(N\right)}$ and integrate for *x*_*d*_, revealing the intended trajectory.

While we treat both the process and actuator as dynamic models in our derivation, it is important to note that both the derivation and the technique are agnostic to the process model, simply because any hypothesis can be modeled and used by our approach to determine where the model would have gone had it not been disturbed.

Note that instead of a dynamic model, any model may be possible, such as lookup table. For instance, a switch is well-modeled as a lookup table or threshold without any consideration of its underlying mechanism. In this case, our technique could be used to determine a person’s intention to flip the switch and how it changes in the face of disturbance. As long as some bidirectional relationship exists between state and outcome (even if determined empirically), intended outcome can be determined. This allows determination of intent in many situations of interest, even where the process is otherwise irreducibly complex.

### Experimental Design

We chose 15 centimeter long simulated reaches of the right arm, beginning at 38 centimeters out from and 5.7 centimeters left of the right shoulder and ending at 38 centimeters out from and 10.7 centimeters right of the right shoulder. We used the following two types of perturbing forces for each combination of distance and direction:

1. Pulse forces applied in one of the the two directions perpendicular to the direction of movement began when the subject had moved either 10% or 50% of the distance to the target and lasted for 150ms.
2. Noise forces began once the subject moved 3 millimeters, and lasted for the duration of the motion. The forces were drawn from a white noise generator at 1000 Hz with flat power spectral density of 1N, and then passed through a 4th order low-pass Butterworth filter with cutoff 10*π* rad/s.

Our error metric was deviation in position, where unsigned error was calculated as the mean magnitude of deviation from the straight-line nominal trajectory to the target. This was evaluated across each of 150 bins measuring 1 millimeter in width and spaced evenly throughout the 15 centimeter reach. Mean unsigned error (MUE) was also used to summarize the overall error in each movement for sensitivity analysis. Mean and maximum signed error relative to the direction of disturbance, also called perpendicular deviation, were used to measure reaching accuracy in order to elucidate direction of any corrections.

### Dynamic simulation of arm and intended trajectories

While the derivation of intent determination is general, testing its application to human reaching requires choosing plant and actuator models, which themselves require physical parameters. Appropriate plant modeling is well-understood: measured arm segment lengths and self-reported body mass are converted into the plant’s inertial, centripetal, and Coriolis terms. Anatomical landmarks and values from Dempster [4] and Winter [5] relate body mass to limb mass, limb length to limb center of mass, and limb mass and length to moment of inertia. For the actuator, we choose the model and parameters of Burdet et al. [6]. We fit a subject-specific constant scale factor for impedance relative to this model to account for any task-dependence of the subjects’ impedance [7] as described in the next section. Desired trajectory in time was idealized as a typical minimum jerk, 5th order polynomial of duration 700 ms starting and ending with zero velocity and acceleration [8]. In this model, musculoskeletal stiffness was linearly related to torque. Like Burdet et al. [6], we approximated muscle torque by looking backward in time 500*μ*s. Note that this backward look led to negligible (sub-micrometer mean unsigned error) discrepancies in subsequent intent estimates.

### Torque Calculations for Intent Extraction

The impedance properties of the human arm are known to be task-dependent [7], but we need to model them in the context of our task without assuming our conclusions in the premises. We do so by scaling an established model [6] using an intermittently and unexpectedly presented secondary task. This ensures that the model parameters are not in any sense tuned to our primary task. Additionally, unpredictability enables us to leverage the ergodic assumption: our tasks should at least initially share the same motor plan and impedance properties.

Calibrating the model requires several steps. First, we fit some expectation of the motor plan, *x*_*d*_, by using a Gaussian weighted average (*σ* = 22 milliseconds) of each subject’s hand trajectory as it evolves in time during undisturbed movement. Separate expectations were fit for each pair of movement start and end points. We then use inverse kinematics to convert these expected hand trajectories to expected joint angle trajectories, *q*_*d*_. Taking time derivatives of *q*_*d*_ then allows us to form an expectation of the feedforward torque, *τ*_*ff*_, that will be present early in movement as in Eq (2),

$$\tau_{ff}=\hat{M}\left(q_{d}\right){\ddot{q}}_{d}+\hat{G}\left(q_{d},{\dot{q}}_{d}\right)$$ (7)

which then allows us to construct the full torque balance for the joints,

$$\tau_{P}+E=\tau_{ff}+c\tau_{fb}$$ (8)

where *E* is the measured force on the hand converted to joint torques via the Jacobian, *τ*_*P*_ is calculated from the arm’s dynamics, *τ*_*fb*_ is calculated from the impedance model of Burdet et al. [6], and *c* is a scalar constant to be fit. We calculate *τ*_*P*_ using measured limb segment lengths, *L*_1_ and *L*_2_ and self-reported body mass, *m*_*g*_. Terms followed by a subscript 1 indicate the upper arm or shoulder joint while terms followed by a subscript 2 indicate the forearm or elbow joint. We converted from these gross measurements to specific parameters using the nominal ratios provided in Table 1,

$$m_{1}=0.028m_{g}\;L_{m1}=0.426L_{1}\;J_{1}=m_{1}{\left(0.322L_{1}\right)}^{2}$$ (9)

$$m_{2}=0.022m_{g}\;L_{m2}=0.682L_{2}\;J_{2}=m_{2}{\left(0.468L_{2}\right)}^{2}$$ (10)

where *m*_1_ and *m*_2_ are segment masses, *L*_*m*1_ and *L*_*m*2_ are limb centers of mass, and *J*_1_ and *J*_2_ are mass moments of inertia. We then calculate *M*, *G*, and *τ*_*P*_ using these subject-specific parameters and measured values for the joint trajectory, *q*, and its time derivatives from reaches that received the white noise force disturbance,

$$M=[\begin{array}{cc} J_{1}+J_{2}+m_{1}L_{m1}^{2}+m_{2}\left(L_{1}^{2}+L_{m2}^{2}+2L_{1}L_{m2}\cos q_{2}\right) & J_{2}+m_{2}\left(L_{m2}^{2}+L_{1}L_{m2}\cos q_{2}\right)\\ J_{2}+m_{2}\left(L_{m2}^{2}+L_{1}L_{m2}\cos q_{2}\right) & J_{2}+m_{2}L_{m2}^{2} \end{array}]$$ (11)

$$G=[\begin{array}{c} m_{2}L_{1}L_{m2}{\dot{q}}_{2}\left(2{\dot{q}}_{1}+{\dot{q}}_{2}\right)\sin q_{2}\\ m_{2}L_{1}L_{m2}{\dot{q}}_{1}^{2}\sin q_{2} \end{array}]$$ (12)

$$\tau_{P}=M\left(q\right)\ddot{q}+G\left(q,\dot{q}\right)$$ (13)

for the first 150 milliseconds following the onset of movement. *τ*_*ff*_ was calculated in the same fashion *τ*_*ff*_ except that $\hat{M}$ and $\hat{G}$ were calculated by replacing all instances of *q* with *q*_*d*_. We then calculated *τ*_*fb*_ as in Burdet et al. [6] while additionally leveraging the dual identity of the muscle torque, *τ*_*m*_,

$$|\tau_{m}\left|=\right|\tau_{ff}+\tau_{fb}\left|=\right|\tau_{P}+E|$$ (14)

$$K=[\begin{array}{cc} 10.8+3.18|\tau_{m1}| & 2.83+2.15|\tau_{m2}|\\ 2.51+2.34|\tau_{m2}| & 8.67+6.18|\tau_{m2}| \end{array}]$$ (15)

$$\tau_{fb}\left(t\right)=K(q_{d}\left(t\right)-q\left(t\right)+\frac{{\dot{q}}_{d}\left(t\right)-q\left(t\right)}{12}+\frac{q_{d}\left(t-\phi \right)-q\left(t-\phi \right)+2\left({\dot{q}}_{d}\left(t-\phi \right)-q\left(t-\phi \right)\right)}{50})$$ (16)

where *ϕ* is a delay of 60 milliseconds. Finally, constrained optimization is performed to find the value of *c* that minimizes the expression,

$$\tau_{ff}+c\tau_{fb}-\tau_{P}-E$$ (17)

for each subject as the sum of all torques acting on the joints should ideally be zero. The optimization is constrained such that *c* > 0.15 both to avoid unphysiological values and numerical instabilities during simulation. The value of *c* is then used when extracting intended trajectories from pulse-disturbed movements as in Eq (3),

$${\ddot{q}}_{d}=\hat{M}{\left(q_{d}\right)}^{-1}\{M\left(q\right)\ddot{q}+G\left(q,\dot{q}\right)+E-\left[\hat{G}\left(q_{d},{\dot{q}}_{d}\right)+c\tau_{fb}\right]\}$$ (18)

and integrated numerically as described. By scaling an established model using a force disturbance other than our disturbance of interest, we avoided presuming the intent trajectory we hoped to recover.

**Table 1:** Arm Parameter Values and Sensitivities.

Parameter Name	Units	Nominal	Source	SD	Source	*S*	*S*_*T*_
Upper Arm Length (*L*_1_)	m	0.353	[4]	0.017	[4]	0.03	0.17
Forearm Length (*L*_2_)	m	0.363	[4]	0.011	[4]	0	0.03
Upper Arm Center of Mass Ratio ($\frac{L_{m1}}{L_{1}}$)	1	0.436	[5]	0.0695	15%	0	0.02
Forearm Center of Mass Ratio ($\frac{L_{m2}}{L_{2}}$)	1	0.682	[5]	0.0431	15%	0.01	0.07
Gross Body Mass (*m*_*g*_)	kg	88.4	[9]	3.1	[10]	0	0.02
Upper Arm Mass Ratio ($\frac{m_{1}}{m_{g}}$)	1	0.028	[5]	0.0029	[4]	0	0.01
Forearm Mass Ratio ($\frac{m_{2}}{m_{g}}$)	1	0.022	[5]	0.0025	[4]	0	0.06
Upper Arm Radius of Gyration Ratio	1	0.322	[5]	0.0161	15%	0.01	0.06
Forearm Radius of Gyration Ratio	1	0.468	[5]	0.0234	15%	0	0.04
Shoulder Parallel Coordinate	m	-0.057	RM	0.02	RM	0.01	0.14
Shoulder Perpendicular Coordinate	m	0.88	RM	0.02	RM	0.02	0.06
Force Sensor Miscalibration *x*-axis	N	0	RM	0.231	RM	0.01	0.05
Force Sensor Miscalibration *y*-axis	N	0	RM	0.1067	RM	0.03	0.08
Force Sensor Gaussian Noise SD *x*-axis	N	0	RM	0.1653	RM	0	0.01
Force Sensor Gaussian Noise SD *y*-axis	N	0	RM	0.2869	RM	0	0.01
Torque-Invariant Impedance Mis-estimation Ratio	1	1	[6]	0.15	15%	0.05	0.19
Torque-Varying Impedance Mis-estimation Ratio	1	1	[6]	0.15	15%	0.05	0.19
Damping-to-Stiffness Ratio (*k*_*d*_)	sec^−1^	0.0833	[6]	0.0125	15%	0.01	0.08
Reflex Impedance Scale Factor	1	0.02	[6]	0.003	15%	0.01	0.04
Reflex Damping to Stiffness Ratio (*g*_*d*_)	sec^−1^	2	[6]	0.3	15%	0	0.04

Synthetic model parameters and their associated mean (nominal) values and standard deviations (SD) used to determine the sensitivity indices of Saltelli et al. [11]. Also shown are the resulting sensitivity indices. Sensitivity indices less than one-thousandth were reported as zero. Total variance was 1.56*mm* ^2^, showing very small average deviations when using this approach. First-order sensitivity, *S*, can be interpreted as fraction of variance removable by perfectly correcting a factor. Total sensitivity, *S*_*T*_, can be interpreted as the fraction left after correcting all other factors. Most parameter values come from the literature, but instrument-specific parameters were determined empirically through repeated measurements (RM). Where parameter values were unavailable, 15% of the mean was used as a conservative estimate.

### Indices of variance-based sensitivity

To ensure a properly spaced and efficient evaluation of sensitivity, we employed Sobol-distributed matrices, generated using MATLAB’s *sobolset()* function and converted to parameter distributions (see Table 1) using inverse cumulative probability density. Nominal parameter values were mostly taken from Dempster [4] and Burdet et al. [6] with typical values for subject-specific parameters (height and weight [9, 10]) and direct measurements for device-specific parameters (force sensor noise and drift). Standard deviations reflect expected variation in repeated measurement (ie. height and weight) or measured variance (ie. trial-to-trial variation in sensor noise or uncertainty in mass ratios reported by Dempster [4]). Distributions were clamped to the range of ±3 standard deviations in order to limit parameters to a realistic range. Combinations and calculations were made as prescribed by Saltelli et al. [11] to arrive at direct and total sensitivity indices.

### Human Subjects

The human data trajectories analyzed here are drawn from eight subjects who gave informed consent in accordance with Northwestern University Institutional Review Board, which specifically approved this study and follows the principles expressed in the Declaration of Helsinki. Five male and three female right-handed subjects (ages 24 to 30) performed the reaches with their right arm and were not compensated. Subjects’ arm segment lengths were directly measured *in situ* while body mass was self-reported.

### Apparatus

A planar manipulandum (described in Patton and Mussa-Ivaldi [12]) was programmed to compensate and minimize any friction or mass. The MATLAB XPC-TARGET package [13] was used to render this force environment at 1000 Hz and data were collected at 1000 Hz. Visual feedback of hand position was performed at 60 Hz using OpenGL. Closed-loop data transmission time (position measurement to completed rendering to recognition of rendering by the position measurement system) was less than 8 milliseconds, ensuring a visual delay less than one 60 Hz frame. Because force sensors tend to drift, we performed a linear re-zeroing procedure between each motion to assure unbiased measurements.

### Protocol

Subjects made 730 reaches in total, along a line parallel to their coronal plane and approximately 45 centimeters from their shoulder. Reaches were either 15 or 30 centimeters long, starting and ending at one of three points spaced 15 centimeters apart on the line. To prevent any learning effect, forces were presented intermittently with random frequency, but never less than 5 reaches apart. Any effects of exposure to pulse forces cannot be detected 5 reaches later [14]. Forces were chosen pseudorandomly such that each type, direction, and distance combination mentioned above was presented 5 times. We used these disturbed, intermittent trials for the analysis.

### Statistical Analysis

Our principal goal was to determine if and when disturbed trajectories departed from undisturbed trajectories. To detect this departure, we use the one-tailed Student’s t-Test (*α* = .05) at 5 millisecond intervals to determine whether or not the deviation of the disturbed trajectories had exceeded the maximum deviations of the undisturbed trajectories. The median time span between departure of the hand and departure of the estimated intent was compared against 120 milliseconds using the nonparametric Sign Test. The MATLAB statistics toolbox package [13] was used for all comparisons.

## Results

**Fig 1:**
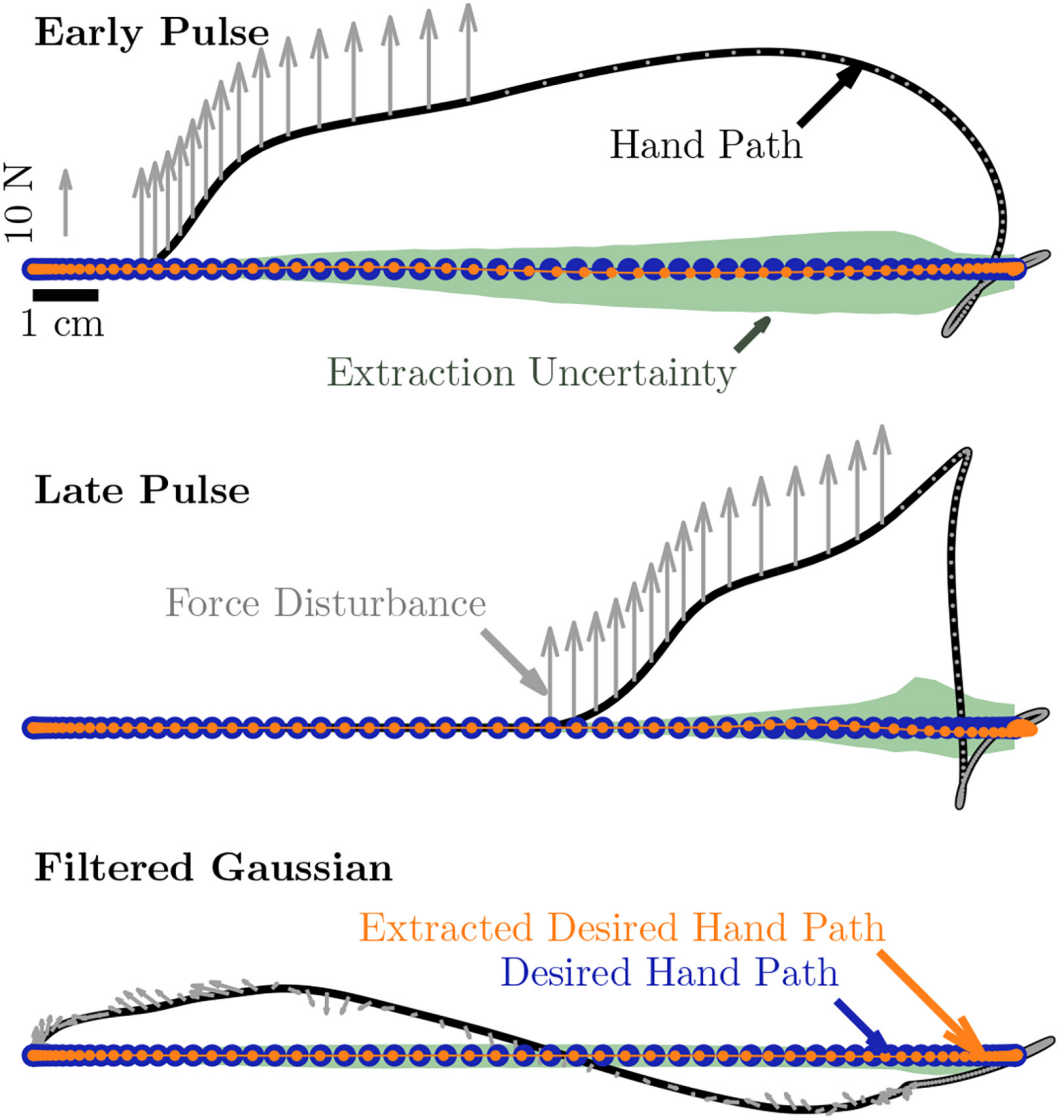
Simulated data illustrating tautology of extraction across pulse and filtered Gaussian noise disturbance types. Intent is modeled as a minimum jerk, 5th order polynomial. Forces experienced are combined with intent via Burdet et al.’s [6] model to produce the simulated arm trajectory. Extraction to recover intention from arm and force trajectory follows. Parameter errors are introduced into the extraction and varied according to Table 1 to estimate sensitivity. Examining the distribution of deviation in trajectories from this analysis reveals that sensitivity is not uniformly distributed throughout a reach.

As expected, the model was able to recover the original intended trajectory even when disturbed (Fig 1). However this idealized analysis cannot reveal any vulnerabilities to unexamined model parameters or to inaccuracy in the structure of the model itself as discussed in the next sections.

### Noise Robustness

While some inversion processes might be highly sensitive to noise, our process for recovering intent from action did not appear to be vulnerable. Our position sensors are very accurate (no detectable drift, RMS noise 1.1 micrometers), but the force sensors are less so. Even after correcting for drift in our force measurements, the force measurements reported during reaching can be expected to have noticeable bias and further noise on the order of that bias as reported in Table 1. Taken together, force measurement errors account for 12% of all sensitivity. We also examined noise in sensor signals explicitly in the more comprehensive sensitivity analysis, discussed below.

### Parameter Sensitivity

Variance in intended trajectories due to estimated uncertainty in model parameters was lower than the natural variation in undisturbed motions. Simulated point-to-point reaches disturbed by either filtered white noise forces or a pulse force perpendicular to the direction of the reach (Fig 1) were extracted in the presence of 220,000 variations upon the parameters (Table 1) according to the methods of Saltelli et al. [11]. Expected variance due to parameter uncertainty was 2.24mm^2^ for pulse forces and.3mm^2^ for filtered white forces. Variance in recorded undisturbed point-to-point reaching under the same time and reach distance conditions was 2.93mm^2^. These variances describe mean unsigned error, but this uncertainty is not distributed evenly in space or time.

**Fig 2:**
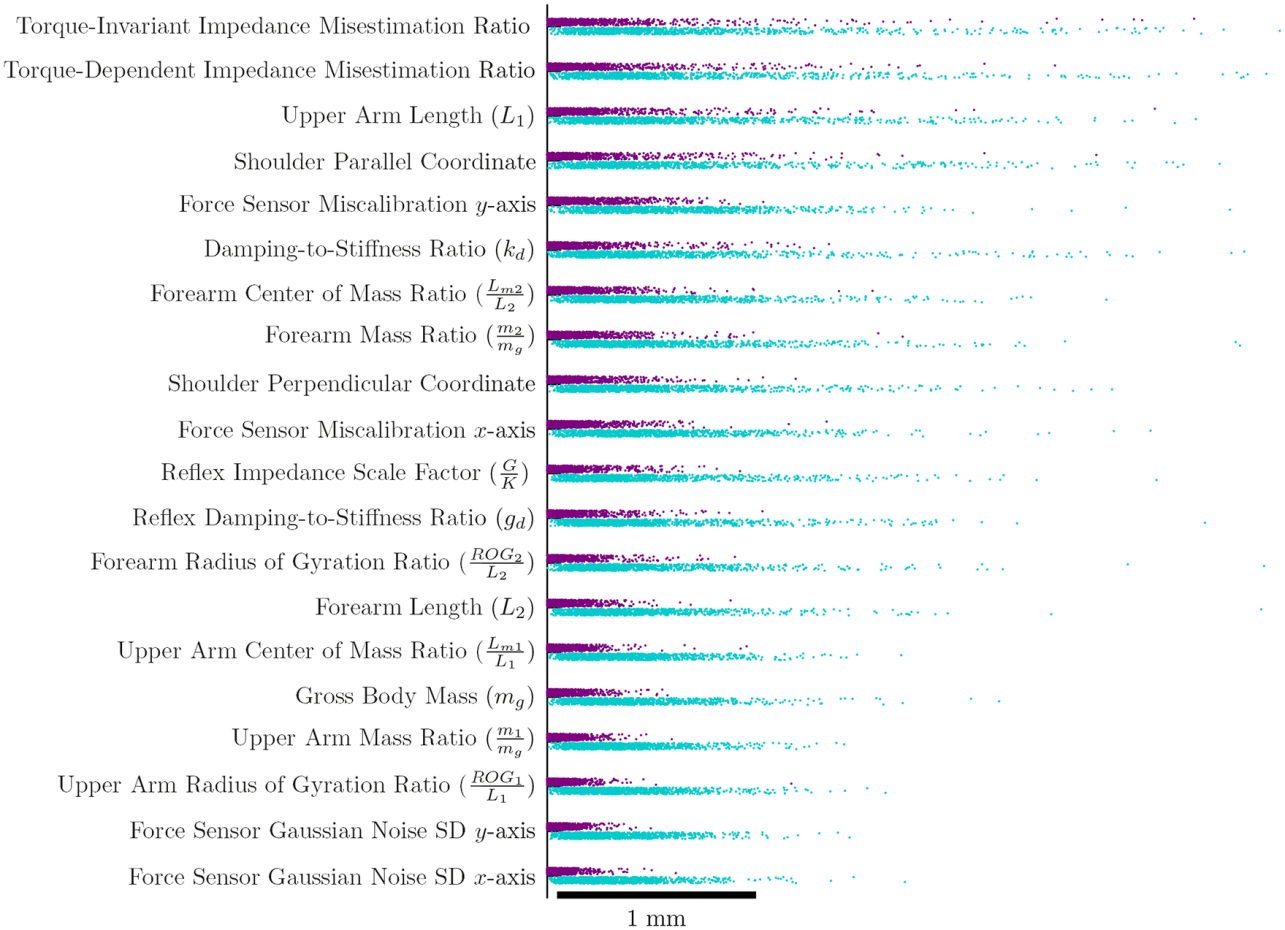
Model sensitivity testing (from the variance-based method of Saltelli et al. [11]) reveals that the model is mostly sensitive to stiffness. Parameter values are varied in a Sobol-distributed fashion according to Table 1. First order sensitivity, in purple, is the error that would be removed if the parameter was fixed at its nominal value. Total sensitivity, in teal, shows error that would remain if all other parameters were fixed at their nominal values.

Simulated error due to direct parameter uncertainty (Fig 2) reached the order of millimeters and revealed particular sensitivity to mis-estimation of stiffness and changes in shoulder position from trial to trial. Direct sensitivity, which reveals the proportion of error that could be removed by correcting inaccuracy of a parameter, was an order of magnitude lower than total sensitivity, which reveals how much error would remain if all other parameters were accurate. Both implicated stiffness estimation inaccuracy as a primary cause of extraction uncertainty. Note that this sensitivity analysis does not provide information on whether models or parameter estimates are accurate, only how sensitive the model would be if they were inaccurate.

### Human Intent Trajectories

**Fig 3:**
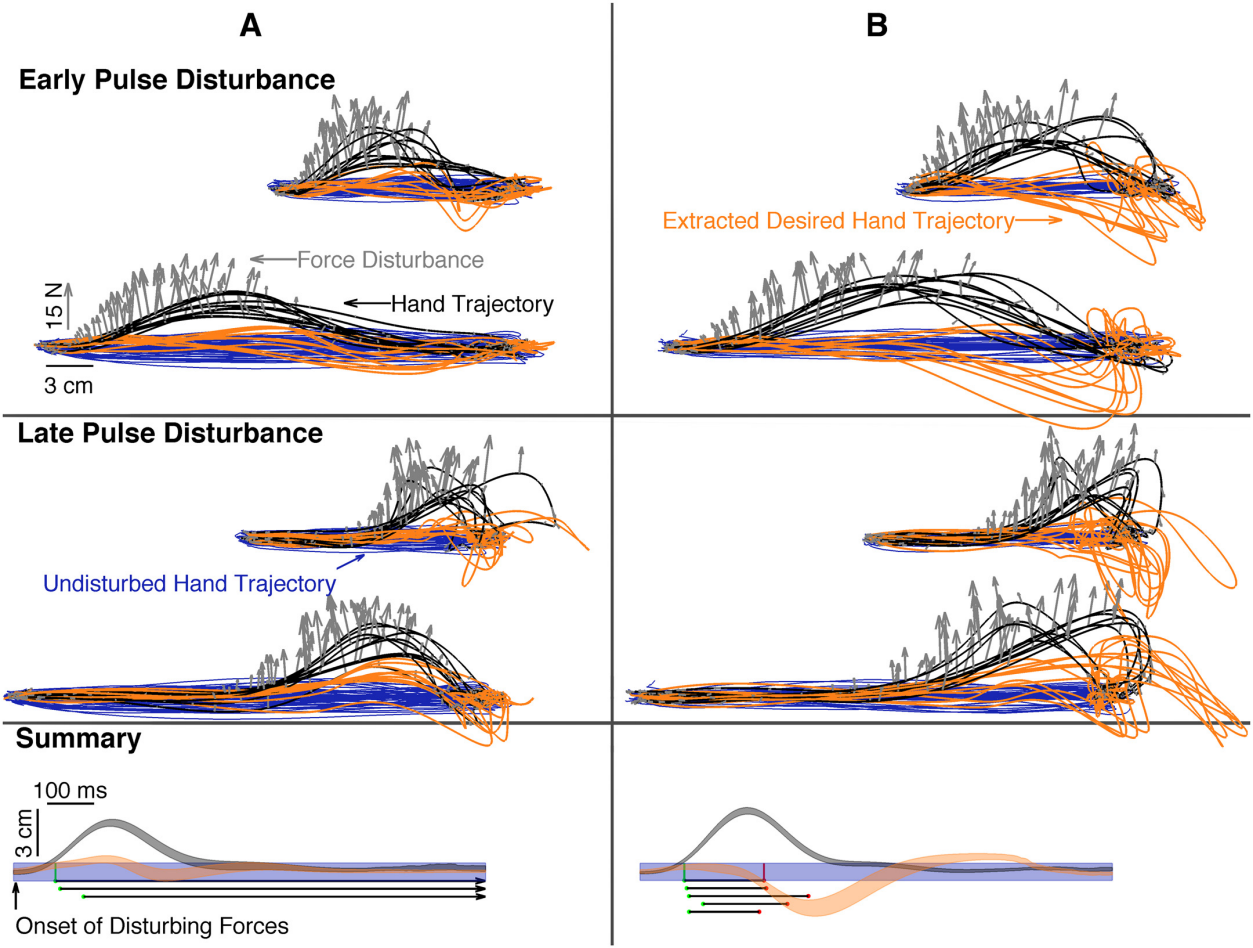
Subjects’ disturbed hand trajectories and extraction of intended trajectories from them reveal that both variance and invariance of the motor plan can occur even in response to very large disturbances. The hand path, in black, deviates from the blue baseline, as a force pulse, gray arrows, is applied. Three of the eight subjects’ intent trajectories (orange) did not significantly deviate from undisturbed movement (panel A) as observed by the intent region not departing the undisturbed movement (blue) region. Five of the eight subjects’ intent trajectories did significantly deviate from undisturbed movement (panel B). The summary plots display whole-subject statistics as shaded regions (mean ± 95% confidence interval on the mean). The time at which the hand region (black) first departed from undisturbed movement (blue) is marked with a green dot for each subject. If the intent region (orange) departed from undisturbed movement, the onset of departure is marked with a red dot for each subject. Panel A subjects’ intent did not depart while panel B subjects’ intents did depart.

Extraction of intent from human point-to-point reaching revealed straight-line movement that persisted for hundreds of milliseconds after the onset of disturbing forces (Fig 3). If intent can change following disturbance, it can only do so after some period of time due to processing and communication delays in the sensorimotor system that produces new motor commands. We hypothesized that intent should not diverge from the undisturbed intent path even if the actual hand was disturbed within this window of delay. In both pulse timing conditions, the intended trajectory remained straight for longer than the hypothesized 120 milliseconds (*p* < 0.001), even though the actual hand was dramatically deflected, supporting our hypothesis and the approach.

These results suggested a reproducible approach for inspecting how individuals might alter their intent in response to disturbances. Actual hand paths could no longer be explained (*p* = 0.05) by deviation in undisturbed movement within 145 milliseconds after the onset of disturbance. Despite the disturbance, three of the eight subjects’ intents did not depart significantly from baseline (Fig 3, panel A). The remaining five subjects’ intents deviated between 150 and 255 milliseconds after the hand deviated (Fig 3, panel B). When disturbances occurred early in reaching, intent showed some signs of correction mid-reach. When disturbances occurred later, the intent continued to the target, but interestingly, it then corrected. Intention showed systematic deflections that counteracted the direction of disturbance beginning about 200 milliseconds after the onset of the force disturbance; however, this counteraction did not immediately depart from the range of undisturbed movements (Fig 3, bottom of panel B).

## Discussion

We sought to test the suitability and robustness of an algorithm that determines a person’s intent during reaching motion, even if there are disturbances. Sensitivity analysis on synthetic data revealed that errors in response to parameter variations were smaller than the trial-to-trial variance commonly observed in human reaching. Mis-estimation was largest if the stiffness was inaccurately modeled. We tested this on human reaching and found that in spite of the hand being disturbed by forces, the intended movement remained on-course to the target, possibly changing after 150 milliseconds. This represents a new and accurate method for viewing intent and how it changes when faced with a force disturbance.

While the derivation of intent determination required many steps, the outcome is a filter whose use is straight-forward. Many filters for converting assumed intent into simulated hand trajectories have been proposed and validated [1, 6, 15]. Demonstrating the validity and plausibility of mathematically transforming those filters to instead convert measured hand trajectories to intent adds a novel method to the arsenal of analytic methods for exploring human motor control.

Interestingly, the formulation leads to some implications on the expected behavior of this moving equilibrium. Even if the muscle equilibrium jumped abruptly, this would be insufficient to move the intended trajectory abruptly due to the presence of the mass term in the intention formulation—reaching intent cannot jump abruptly. The intent trajectories certainly follow this pattern, smoothly responding to disturbances at a latency of about 200 milliseconds. While this work examines the tendencies of the onset of change, statistical testing cannot be used to detect the absence of change in an individual movement. Still, Fig 3 shows reaches that are more compatible with intermittent control than with continuous optimal feedback control. In classic optimal control, the system is always updating intent in response to error [16]. While most of our subjects displayed a latent response consistent with sensory feedback latencies [17], three of our eight subjects showed no detectable response. The absence of response to a large force disturbance is instead consistent with adherence to a trajectory planned (perhaps entirely) before the disturbance. Control that is intermittent [18] with limited opportunities to change intent can explain such observations. It remains to be seen if and how change in intent might be triggered.

Determination of intent may facilitate motor training and stroke recovery. Error augmentation, which presently relies on dictating the reaching intent, has demonstrated the capacity to increase and speed up learning in healthy patients [12] and following stroke [19]. Augmentation of difference from static, dictated intent could be replaced with scaling of the magnitude of the difference between the desired and realized trajectory. This makes error augmentation during undirected reaching and exploration possible, including both error reduction and error magnification. Partial error cancellation would allow learning to take place without harm to task goals. As demonstrated by the success of the challenge-point framework [20], dynamic variation of augmentation as task learning progresses can be beneficial. With this extraction, error can be measured and augmented in real-time even without an explicit task, potentially enabling wider utility and application.

This novel determination of intended trajectories from force-disturbed movements allows hypothesis testing that was not previously possible. This approach can take **any** “candidate model” of the human arm and create a filter that estimates the intended trajectory. By using tests such as the comparison with undisturbed straight line reaching employed in this paper, one can evaluate which candidate produces the most plausible intended trajectory, supporting one model over another. Instead of comparing two generative models to see which better approximates movement, it is now possible to perform element-by-element fitting. Direct access to the intended trajectory allows optimal feedback control models to better understand and fit hypothesized cost functions and their parameters. In addition, mid-movement replanning became falsifiable: we were able to show that intended trajectories from disturbed motions differ from those of undisturbed motions and how they differed.

Nevertheless, there are some limitations to these methods. Accurate interface force measurement (within a few tenths of a newton) is needed. Such instrumentation is available, but must be used with great care in order to preserve the accuracy of the estimate. Our sensitivity analysis on synthetic data revealed that slight error can lead to a trajectory that accumulates a drift due to bias caused by the force term. More importantly, while modern force sensors can be highly accurate with very high signal to noise ratios, they tend to drift over time, leading to error that can grow if the device is not periodically tared. Our empirical approach used several methods to mitigate these effects, and hence the errors became small. It remains to be seen if there are methods to further reduce the mis-estimation due to force error or eliminate the need for force estimation altogether. It will be also interesting to determine whether relaxing constraints and operating in 3D would produce the same levels of accuracy found here.

Since trajectories were not controlled experimentally, our tests rely heavily on the assumption that disturbed intentions initially mirror undisturbed intentions. This study examined straight line motions because it is well understood that people tend to repetitively attempt to reach in a straight line towards a target [21]. Also, even when subjects are persistently perturbed by a force field, they recover their motions to a straight line with repetitive experience [1]. Future studies might employ tasks with a more specific and/or explicit trajectory to facilitate comparison with the estimate intent’s path. Unfortunately, even such an intended trajectory may not be strictly invariant during external disturbance because some displacements may cause intent to be recalculated by the nervous system.

Another key assumption in this study is that the joint impedance (stiffness) is linearly related to torque. While other approaches tend to construct paradigms that indicate or presume that the intention is static in order to derive impedance—the so called family of system identification applications, this study makes a constrained assumption of stiffness in order to determine intent. In a sense, this approach widens the scope to include movement. Sensitivity analyses showed that approximation of stiffness is the most critical factor to accuracy in estimating intent. While serious trial-to-trial stiffness mis-estimation is unlikely, co-contraction in response to disturbance might plausibly double stiffness mid-reach. Even as the model is insensitive to normal variations, a human can halve or double their stiffness [22] significantly reducing determination accuracy. Our results also point to the possibility of the impedance changing during motion, perhaps as a function of time. This stiffening may even explain the reported deviation of intent from the baseline trajectories. Of particular concern are the large hooks at the end of the motions. While subjects might have preemptively stiffened in response to the possibility of disturbance, the many reaches in between disturbances may have induced them to relax instead. These changes in stiffness might be substantiated (or even corrected for) by detecting muscle co-contraction using electromyography or using additional modeling assumptions to allow real-time estimation of stiffness.

The simple linear scaling of Burdet et al.’s stiffness model [6] facilitated hypothesis testing, but was inherently limiting. However, more accurate approaches would provide us with many free parameters through which to determine the outcome of the eventual extraction and obtain a spurious favorable result. Like others who have examined large force disturbances, we find lower stiffnesses (our *c* parameter) than might be expected [23] (S1 Table). We explored the possibility that both the hooks and the low stiffness resulted from underestimating the contribution of reflex impedance, but using values more similar to those of Crevecouer and Scott [23] exacerbated the hooks without increasing total stiffness. Differences in tasks studied [7] and/or the dependency of muscle impedance on perturbation size [24] may explain these discrepancies.

The general intent extraction approach presented here reveals the conditions needed to solve for the moving intent in an arbitrary dynamic process. Sensitivity analysis for this approach applied to the human arm demonstrated favorable conditions for determination of intent during force-disturbed reaching. Reaching intent became significantly different from baseline reaching only after enough time had elapsed for the disturbance to be processed and descending motor commands to change. This window into intent should allow advances in arm modeling, motor training, and human-machine interaction.

## Supporting Information

S1 Table Subject-specific parameters. Gross body mass was self-reported. Upper arm length, forearm length, and shoulder position in the robot’s coordinate system were measured *in situ*. Feedback torque gain was fit as described in our methods section using the intermittently presented white noise force disturbance. This gain is relative to the total feedback torque output (stiffness, damping, and reflexes) of the model of Burdet et al. [6].
(PDF)

S1 Text What is intent. (PDF)
