# Supplementary material for: I Meant to Do That: Determining the Intentions of Action in the Face of Disturbances
Source: PLoS One. 2015 Sep 1;10(9):e0137289. doi: 10.1371/journal.pone.0137289 (PMC4556620; doi:10.1371/journal.pone.0137289)
Supplement: S1 Table — Gross body mass was self-reported. Upper arm length, forearm length, and shoulder position in the robot’s coordinate system were measured in situ. Feedback torque gain was fit as described in our methods section using the intermittently presented white noise force disturbance. This gain is relative to the total feedback torque output (stiffness, damping, and reflexes) of the model of Burdet et al. [6]. (PDF) [file pone.0137289.s001.pdf]

Table S1. Subject-Specific Measured and Fit Parameters

Subject Number	1	2	3	4	5	6	7	8
Gross Body Mass (kg), m_g	86.18	54.43	95.25	86.18	94.35	86.18	63.50	72.57
Upper Arm Length (cm), L_1	28	28	31	33	34	37	28	29
Forearm Length (cm), L_2	32	30	34	34.5	34	38	31	33
Shoulder Parallel Coordinate (cm)	0	-3	0	0	-2	-3	-5	0
Shoulder Perpendicular Coordinate (cm)	53	48	51	49	55	51	48	50
Feedback Torque Gain, c	0.15	0.15	0.36	0.15	0.94	0.77	0.48	0.86
