# Supplementary material for: I Meant to Do That: Determining the Intentions of Action in the Face of Disturbances
Source: PLoS One. 2015 Sep 1;10(9):e0137289. doi: 10.1371/journal.pone.0137289 (PMC4556620; doi:10.1371/journal.pone.0137289)
Supplement: S1 Text — (PDF) [file pone.0137289.s002.pdf]

The word *intent* can be contentious and lead to confusion; and hence may be best placed in the context of various literature that use related terminology. It is important to distinguish intended action from *motivation* [1, 2], *cost* [3, 4], or *goal selection decisions* [5]. In typical motor control studies, subjects are motivated to complete an experiment in a timely fashion, and are usually explicitly provided targets to reach to. Classification of intent is prevalent in both lower [6, 7] and upper limb [8, 9] prosthetics where hybrid control algorithms select from among a set of discrete actions (walking/standing/flexion/extension/etc). While subjects may be *motivated* to complete experiments with minimal effort/cost and their *goal* may be to reach a target, here we use intent to describe the course of action (i.e., the trajectory of the arm) taken in service of goals and motives and not the goals nor the motives themselves. Particularly of interest is the intended course of action (i.e, the intended trajectory), even when the actual movement is disturbed and hence no longer matches the intent. In other words, we operationally define *intent* as a subjunctive – where would the motion have gone had it not been disturbed?

Attempts to deduce motor intent in the past have focused on the assumed spring-like properties of human muscles. Springs produce a force according to their impedance and stretch. By measuring force, impedance, and position, Gomi and Kawato [10] were able to deduce stretch and thereby infer the muscle's equilibrium point. Supporters of the "λ model" [11] hypothesized that this muscle equilibrium point, and not the equilibrium of the whole arm, represented the intent of a movement even though it did not compensate for the dynamics of the arm as a feedforward controller would. Upon Gomi and Kawato's inspection of the muscle equilibrium point as it evolved in time, it was clear that it was highly complex and often not anatomically realizable. Therefore, it could not well-represent the intent of a simple reaching movement. Unlike the equilibrium point of muscle, the a pre-planned equilibrium point of the entire arm is the path the arm will follow in the absence of disturbance [12]. In the presence of disturbance, the arm might be deflected from its equilibrium. We explored if or how this arm equilibrium point might change due to disturbances.

Our findings could explain the discovery by Bizzi et al. [13] of a "virtual trajectory" (α) in deafferented monkeys that progressed smoothly from the initial to final positions and how that virtual trajectory is different from λ, the equilibrium point of muscles – intent and λ are equivalent only when intent is unchanging; therefore, this muscle equilibrium cannot be the intent of a movement. By deriving the technique in a general form, we discovered that our intent is equivalent to the virtual trajectory and can be used to determine the muscle equilibrium of a λ model (See Methods section).

References

1. McClelland DC (1985) How motives, skills, and values determine what people do. American Psychologist 40: 812.

2. Rawolle M, Schultheiss M, Schultheiss OC (2013) Relationships between implicit motives, self-attributed motives, and personal goal commitments. Frontiers in psychology 4.

3. Todorov E, Jordan MI (2002) Optimal feedback control as a theory of motor coordination. Nature neuroscience 5: 1226–1235.

4. Flash T, Hogan N (1985) The coordination of arm movements: an experimentally confirmed mathematical model. The journal of Neuroscience 5: 1688–1703.

5. Ziebart BD, Bagnell J, Dey AK (2010) Modeling interaction via the principle of maximum causal entropy. In: Proceedings of the 27th International Conference on Machine Learning (ICML-10). pp. 1255–1262.

6. Strausser KA, Kazerooni H (2011) The development and testing of a human machine interface for a mobile medical exoskeleton. In: Intelligent Robots and Systems (IROS), 2011 IEEE/RSJ International Conference on. IEEE, pp. 4911–4916.

7. Hargrove LJ, Simon AM, Young AJ, Lipschutz RD, Finucane SB, et al. (2013) Robotic leg control with emg decoding in an amputee with nerve transfers. New England Journal of Medicine 369: 1237–1242.

8. Englehart K, Hudgins B (2003) A robust, real-time control scheme for multifunction myoelectric control. Biomedical Engineering, IEEE Transactions on 50: 848–854.

9. Young AJ, Hargrove LJ, Kuiken TA (2012) Improving myoelectric pattern recognition robustness to electrode shift by changing interelectrode distance and electrode configuration. Biomedical Engineering, IEEE Transactions on 59: 645–652.

10. Gomi H, Kawato M (1997) Human arm stiffness and equilibrium-point trajectory during multi-joint movement. Biological cybernetics 76: 163–171.

11. Feldman AG, Levin MF (1995) The origin and use of positional frames of reference in motor control. Behavioral and Brain Sciences 18: 723–744.

12. Shadmehr R, Mussa-Ivaldi FA (1994) Adaptive representation of dynamics during learning of a motor task. The Journal of Neuroscience 14: 3208–3224.

13. Bizzi E, Accornero N, Chapple W, Hogan N (1984) Posture control and trajectory formation during arm movement. The Journal of Neuroscience 4: 2738–2744.
